# Extracellular vesicles from *Akkermansia muciniphila* protect streptozotocin-induced diabetic mice by regulating glucose homeostasis, oxidative stress, and immune tolerance

**DOI:** 10.3389/fimmu.2026.1739048

**Published:** 2026-04-07

**Authors:** Yunxiang Chang, Cheng Zhang, Zhipeng Li, Shikai Wang, Jialin Wu, Jiacheng Xie, Xinsheng Cheng

**Affiliations:** Department of Hepatobiliary Surgery, Shenzhen Nanshan People’s Hospital, Shenzhen, Guangdong, China

**Keywords:** *Akkermansia muciniphila*, extracellular vesicles, pancreatic immune, regulatory T cell, type 1 diabetes mellitus

## Abstract

**Introduction:**

Accumulating evidence suggests that extracellular vesicles (EVs) derived from gut microbiota play important roles in modulating host metabolism and immune responses, thereby influencing the development of metabolic disorders including diabetes mellitus. *Akkermansia muciniphila* (*A. muciniphila*), a mucin-degrading bacterium, exerts beneficial effects on glucose metabolism and immune regulation.

**Methods:**

This study investigated the protective effects of *A. muciniphila*-derived extracellular vesicles (AmEVs) in streptozotocin (STZ)-induced type 1 diabetes mellitus (T1DM) mice. Metabolic parameters, pancreatic histology, oxidative stress, inflammatory cytokines, and regulatory T cell (Treg) responses were assessed. Anti-CD25–mediated Treg depletion was performed to evaluate the functional role of Tregs.

**Results and discussion:**

STZ-induced T1DM mice exhibited a significantly reduced abundance of *A. muciniphila*. AmEV treatment ameliorated hyperglycemia and improved glucose tolerance, insulin tolerance, and pyruvate tolerance, showing greater metabolic improvement than heat-inactivated *A. muciniphila*. AmEVs preserved pancreatic islet morphology and enhanced β-cell function, accompanied by improved systemic metabolic parameters. In addition, AmEVs reduced oxidative stress and suppressed inflammatory cytokine production while enhancing Treg-associated immunoregulation in pancreatic tissue. Importantly, anti-CD25–mediated Treg depletion partially reversed the metabolic and anti-inflammatory benefits of AmEV treatment. These findings suggest that AmEVs alleviate diabetic pathology through coordinated metabolic and immune regulation. The protective effects are at least partly dependent on Treg-mediated immunoregulation, highlighting the potential of AmEVs as microbiota-derived therapeutic candidates for T1DM.

## Introduction

Type 1 diabetes mellitus (T1DM) is a chronic autoimmune disease caused by the immune-mediated destruction of insulin-producing pancreatic β-cells (1), leading to insulin deficiency and persistent hyperglycemia. It typically develops in children or young adults and requires lifelong insulin therapy (2). Although insulin replacement can delay disease progression, it does not address the underlying autoimmune processes or prevent long-term complications such as nephropathy, retinopathy, and cardiovascular disease (3, 4). Increasing evidence suggests that immune dysregulation, particularly within the pancreatic microenvironment, plays a central role in T1DM onset and progression (5). A deeper understanding of the immune mechanisms involved in β-cell destruction has opened avenues for therapeutic interventions that aim to restore immune balance rather than merely control blood glucose.

One of the key immunological mechanisms underlying β-cell destruction is the breakdown of peripheral immune tolerance (6). In the pancreatic microenvironment, this breakdown facilitates the activation of autoreactive T cells that target islet antigens (7). Regulatory T cells (Tregs), characterized by the expression of Foxp3, play a central role in maintaining immune homeostasis by suppressing excessive immune activation and promoting self-tolerance. In T1DM, a reduction in Treg numbers or suppressive function has been consistently observed both in patients and in animal models (8–10). Enhancing Treg responses and restoring immune tolerance within the pancreatic islets are thus considered promising strategies for modulating immune dysregulation associated with T1DM.

Meanwhile, increasing attention has been paid to the gut microbiota as a potential modulator of both metabolic and immune pathways relevant to T1DM (11). Perturbations in microbial composition have been linked to increased intestinal permeability (12), systemic inflammation (13), and altered T-cell differentiation (14). Among the gut microbial taxa, *Akkermansia muciniphila* (*A. muciniphila*), a mucin-degrading bacterium residing in the intestinal mucus layer, has emerged as a key player in host–microbe interactions (15). Reduced abundance of *A. muciniphila* has been reported in multiple disorders, including pre-eclampsia (16), hepatic encephalopathy (17), and diabetes (18). Experimental studies have shown that supplementation with *A. muciniphila* can improve glucose homeostasis (19), strengthen gut barrier integrity (20), and modulate host immunity (21), suggesting its potential as a next-generation probiotic.

Beyond live bacteria, recent studies have revealed that bacteria-derived extracellular vesicles (EVs) serve as critical mediators of microbe-host communication (22). EVs are nano-sized membrane-bound particles secreted by both Gram-negative and Gram-positive bacteria, capable of delivering bioactive molecules to host cells and influencing distant organ functions (23). EVs from *A. muciniphila* (AmEVs) have shown promising potential in various disease models, such as hypertension (24), colitis (25), and so on; and other inflammatory disorders, and in some cases have demonstrated superior efficacy compared with the bacterium itself (26, 27). More importantly, the critical role of *A. muciniphila* in host immune regulation has been highlighted in recent years. For example, *A. muciniphila* has been reported to mediate the negative effects of interferon-γ (IFN-γ) on glucose metabolism (28), and to induce gut microbiota remodeling and control islet autoimmunity in non-obese diabetic (NOD) mice (29). However, whether AmEVs exert therapeutic benefits in autoimmune diabetes remains unknown. Moreover, their role in regulating pancreatic immune tolerance, especially through the modulation of Treg populations, has not been systematically investigated.

To address this gap, the present study aimed to investigate the protective effects of AmEVs in a streptozotocin (STZ)-induced T1DM mouse model, with a particular focus on glucose metabolism and pancreatic immune regulation. Although the STZ model primarily reflects β-cell injury–driven diabetes rather than spontaneous autoimmune disease, it is accompanied by significant inflammatory and immune perturbations, allowing evaluation of immunomodulatory interventions under diabetic inflammatory conditions. These findings may help clarify the therapeutic potential of bacterial vesicles in autoimmune diabetes and support the development of microbiota-derived interventions.

## Materials and methods

### Study approval

The Animal Research Committee of the Shenzhen Nanshan People’s Hospital approved the animal experiments, ensuring full compliance with the institution’s guidelines for animal care during housing and handling. The study protocol was approved by the Institutional Animal Care and Use Committee (IACUC) of Shenzhen Nanshan District People’s Hospital (Approval No.: A202400918; approval date: 18 September 2024). All procedures were conducted in accordance with national and institutional guidelines and were reported in compliance with the ARRIVE 2.0 guidelines.

Mice were monitored daily for general health status and signs of distress. Humane endpoints included more than 20% body weight loss, severe lethargy, or inability to access food and water. All efforts were made to minimize animal suffering. Euthanasia was performed under deep isoflurane anesthesia followed by cervical dislocation.

After confirmation of hyperglycemia, mice were randomly assigned to experimental groups using a computer-generated randomization sequence. Investigators responsible for metabolic testing, histological quantification, and data analysis were blinded to group allocation. Mice that did not reach the predefined hyperglycemic criterion (Fasting blood glucose, FBG ≥11.1 mM) were excluded before randomization. No animals were excluded after group allocation. Group allocation codes were maintained by an independent investigator until completion of data analysis.

### Animal husbandry and T1DM model induction

Male C57BL/6 mice (5–6 weeks old, 18-22g) were purchased from Guangdong Medical Laboratory Animal Center and housed in a specific pathogen-free facility under standard conditions (12 h light/dark cycle, 22 ± 2 °C, 55 ± 10% humidity) with *ad libitum* access to food and water. Environmental enrichment, including nesting materials and shelters, was provided throughout the study period. After one week of acclimatization, T1DM was induced by intraperitoneal injection of STZ (50 mg/kg/day, dissolved in 0.1 mol/L citrate buffer, pH 4.5) for five consecutive days. FBG levels were measured 72 h after the final injection, and mice with FBG ≥11.1 mM were considered T1DM.

### Fecal collection and quantification of A. muciniphila

Fresh fecal samples were collected from individual mice at week 10, snap-frozen in liquid nitrogen, and stored at -80 °C. Genomic DNA was extracted using the E.Z.N.A.^®^ Stool DNA Kit (Omega Bio-tek). The relative abundance of *A. muciniphila* was determined by quantitative PCR using specific primers (5’-CAGCACGTGAAGGTGGGGAC, AM2: 5’-CCTTGCGGTTGGCTTCAGAT). Universal bacterial primers (UniF340/UniR514: 5’-ACTCCTACGGGAGGCAGCAGT/5’-ATTACCGCGGCTGCTGGC) were used as internal control.

### Preparation and characterization of AmEVs

*A. muciniphila* (ATCC BAA-835) was anaerobically cultured in mucin-based medium. Bacterial culture supernatants were sequentially filtered through 0.45 µm and 0.22 µm membranes, followed by ultracentrifugation at 150,000 × g for 2 h at 4 °C. The obtained EVs (AmEVs) were washed and resuspended in sterile PBS. Morphology was assessed by transmission electron microscopy (TEM), and particle size and concentration were determined using nanoparticle tracking analysis (NTA).

### Animal grouping, treatment, and sample collection

After STZ administration, FBG was measured 48 hours after the final injection. Mice with FBG ≥11.1 mM were considered T1DM and selected for subsequent treatment. Mice were then randomly divided into six groups (n = 6 per group): (1) Control (non-diabetic, no STZ), (2) T1DM (STZ-induced, untreated), (3) T1DM + AKK.m, (4) T1DM + AmEV low dose (AmEV-L), (5) T1DM + AmEV medium dose (AmEV-M), (6) T1DM + AmEV high dose (AmEV-H). Mice in the T1DM + AKK.m group received oral gavage of 10^5^ CFU heat-inactivated *A. muciniphila* suspended in 100 μL saline once daily. The T1DM + AmEV groups received 10 μg (low), 20 μg (medium), or 40 μg (high) of AmEVs in 100 μL saline per day via oral gavage. Control and T1DM mice received 100 μL sterile saline daily as vehicle control. All treatments were administered continuously for 8 weeks.

At the end of the intervention, mice were euthanized during the final week. Blood samples were collected by cardiac puncture and centrifuged to obtain serum. Pancreatic and hepatic tissues were excised, weighed, and processed for histological staining, protein analysis, or snap-frozen in liquid nitrogen for subsequent assays.

### Body weight monitoring and metabolic tests

Body weight was recorded weekly after experimental beginning. Glucose metabolism was assessed using oral glucose tolerance test (OGTT), insulin tolerance test (ITT), and pyruvate tolerance test (PTT). For OGTT and PTT, mice were fasted for 12 h and injected orally with glucose (2 g/kg) or intraperitoneally with pyruvate (2 g/kg), respectively. For ITT, mice were fasted for 6 h and injected intraperitoneally with insulin (0.75 U/kg). Blood glucose was measured from tail vein at 0, 15, 30, 60, 90, and 120 min post-administration.

### Measurement of FBG, fasting insulin, and β-cell function

FBG was measured using Glucose Assay Kit (Sigma, cat.no MAK476) while fasting serum insulin (FINS) was quantified by enzyme-linked immunosorbent assay (ELISA) (Aviva Systems Biology, cat.no OKRC01234). β-cell function was assessed using the HOMA-β index: HOMA-β = 20 × FINS/(FBG – 3.5).

### Histological analysis and islet morphometry

Pancreatic and hepatic tissues were fixed in 4% paraformaldehyde for 24–48 hours at room temperature, embedded in paraffin, and sectioned at 4 μm thickness. Sections were stained with hematoxylin and eosin (H&E) following standard protocols. For pancreatic tissue, islet morphology and area were evaluated under a light microscope. Quantification of islet area was performed using ImageJ software by analyzing at least 10 randomly selected islets per mouse from multiple pancreatic sections, and the mean islet area per mouse was used for statistical analysis. Histological evaluation and morphometric analysis were performed by investigators blinded to group allocation.

For hepatic tissue, histopathological changes including hepatocyte ballooning, steatosis, and inflammatory infiltration were assessed. Representative images were captured and liver architecture integrity was qualitatively compared across groups to evaluate treatment-related improvements.

### Western blot analysis for insulin expression

Total protein was extracted from pancreatic tissue and quantified by BCA assay. Equal amounts of protein were separated by SDS-PAGE, transferred to PVDF membranes, and probed with anti-insulin (Abcam, cat.no ab181547) and anti-β-actin (Beyotime, cat.no AF5003) antibodies. Bands were visualized using ECL substrate and analyzed by densitometry with ImageJ software.

### Assessment of liver function and lipid metabolism

Serum levels of alanine aminotransferase (ALT), aspartate aminotransferase (AST), and triglycerides (TG) were measured using the corresponding commercial kits (ALT, Abcam, cat.no ab285263; AST, elkbiotech, cat.no ELK1778; TG, Abcam, cat.no ab65336).

### Evaluation of oxidative stress markers

Levels of oxidative stress in serum and pancreatic tissues were assessed by measuring malondialdehyde (MDA), glutathione peroxidase (GSH-Px), superoxide dismutase (SOD), and catalase (CAT) using commercially available colorimetric assay kits (MDA, Beyotime, cat.no S0131S; GSH-Px, Abcam, cat. no ab102530; SOD, Solarbio, cat. no BC0175; CAT, Solarbio, cat.no BC0205). Sample preparation and assay procedures were performed in accordance with the manufacturer’s instructions.

### Inflammatory cytokine analysis

The levels of inflammatory cytokines including tumor necrosis factor-α (TNF-α), interleukin-6 (IL-6), IFN-γ, and interleukin-1β (IL-1β) were measured in serum and pancreatic tissues using ELISA kits (TNF-α, Thermo Fisher, cat.no BMS607-2HS; IL-6, Thermo Fisher, cat. no BMS603HS; IFN-γ, Cell Sciences, cat. no CKM036; IL-1β, Abcam, cat.no ab229440). For tissue samples, protein was extracted from frozen pancreatic tissue using RIPA buffer containing protease inhibitors, and total protein concentrations were determined by the BCA method. Cytokine levels in tissues were normalized to total protein content.

### Flow cytometric analysis of Tregs in pancreatic lymph nodes

Single-cell suspensions from pancreatic draining lymph nodes were prepared for surface staining with fluorochrome-conjugated antibodies against CD3 (PerCP/Cyanine5.5, clone 17A2, BioLegend, catalog no. 100218), CD4 (FITC, clone RM4-5, BioLegend, catalog no. 100510), and CD25 (APC, clone PC61, BioLegend, catalog no. 102012). After surface staining, cells were fixed and permeabilized using the Foxp3/Transcription Factor Staining Buffer Set (Invitrogen, catalog no. 00-5523-00) according to the manufacturer’s instructions. Intracellular staining was then carried out using anti-Foxp3 (PE, clone FJK-16s, Invitrogen, catalog no. 12-5773-82) antibody. Flow cytometry was performed on a BD FACSCanto II system, and data were analyzed using FlowJo software. At least 50,000 events were acquired for each sample. Gating strategy first identified lymphocytes based on FSC-A and SSC-A parameters, followed by doublet exclusion using FSC-A versus FSC-H gating. CD3^+^ T cells were then gated, followed by identification of CD4^+^ T cells, followed by CD25^+^Foxp3^+^ cells within the CD3^+^CD4^+^ population to define Tregs. Representative gating strategies are shown in **Supplementary Figure 1**.

### Immunofluorescence detection of Tregs in the pancreas

Paraffin-embedded pancreatic sections were deparaffinized and subjected to heat-mediated antigen retrieval using citrate buffer (pH 6.0). Sections were incubated with 5% bovine serum albumin for 1 h at room temperature and incubated overnight at 4 °C with primary antibodies against Foxp3 (rabbit anti-Foxp3, Servicebio, catalog no. GB112325, dilution 1:2500) and CD4 (rabbit anti-CD4, Servicebio, catalog no. GB15064, dilution 1:2000). After washing, sections were incubated with Alexa Fluor–conjugated secondary antibodies (goat anti-rabbit Alexa Fluor 555 and goat anti-rabbit Alexa Fluor 488, dilution 1:500) for 1 h at room temperature in the dark. Nuclei were counterstained with DAPI. Images were captured using a fluorescence microscope (model, manufacturer) under identical exposure settings for all groups.

### Treg depletion experiment

Anti-CD25 antibody (clone PC61) was used to deplete CD25^+^ T cells *in vivo*. Mice received intraperitoneal injections of anti-CD25 antibody prior to AmEV treatment, followed by maintenance injections every three weeks. Depletion efficiency was confirmed by flow cytometric analysis of CD4^+^Foxp3^+^ T cells in pancreatic draining lymph nodes.

### Statistical analysis

Data are presented as mean ± SEM. Time-course data, including body weight, OGTT, ITT, and PTT curves, were analyzed using two-way repeated-measures ANOVA followed by Tukey’s multiple comparisons test. For endpoint comparisons among multiple groups, one-way ANOVA followed by Tukey’s *post hoc* test was used. The area under the curve (AUC) for OGTT, ITT, and PTT was calculated using the trapezoidal rule based on glucose measurements at each time point. Before applying parametric statistical tests, normality was assessed using the Shapiro–Wilk test, and variance homogeneity was evaluated. For analyses involving multiple cytokines and oxidative stress markers, false discovery rate (FDR) correction using the Benjamini–Hochberg method was applied. A p-value < 0.05 was considered statistically significant. GraphPad Prism 9.0 was used for all analyses.

## Results

### STZ-induced T1DM mice showed a decrease in the abundance of A. muciniphila levels

**Figure 1:**
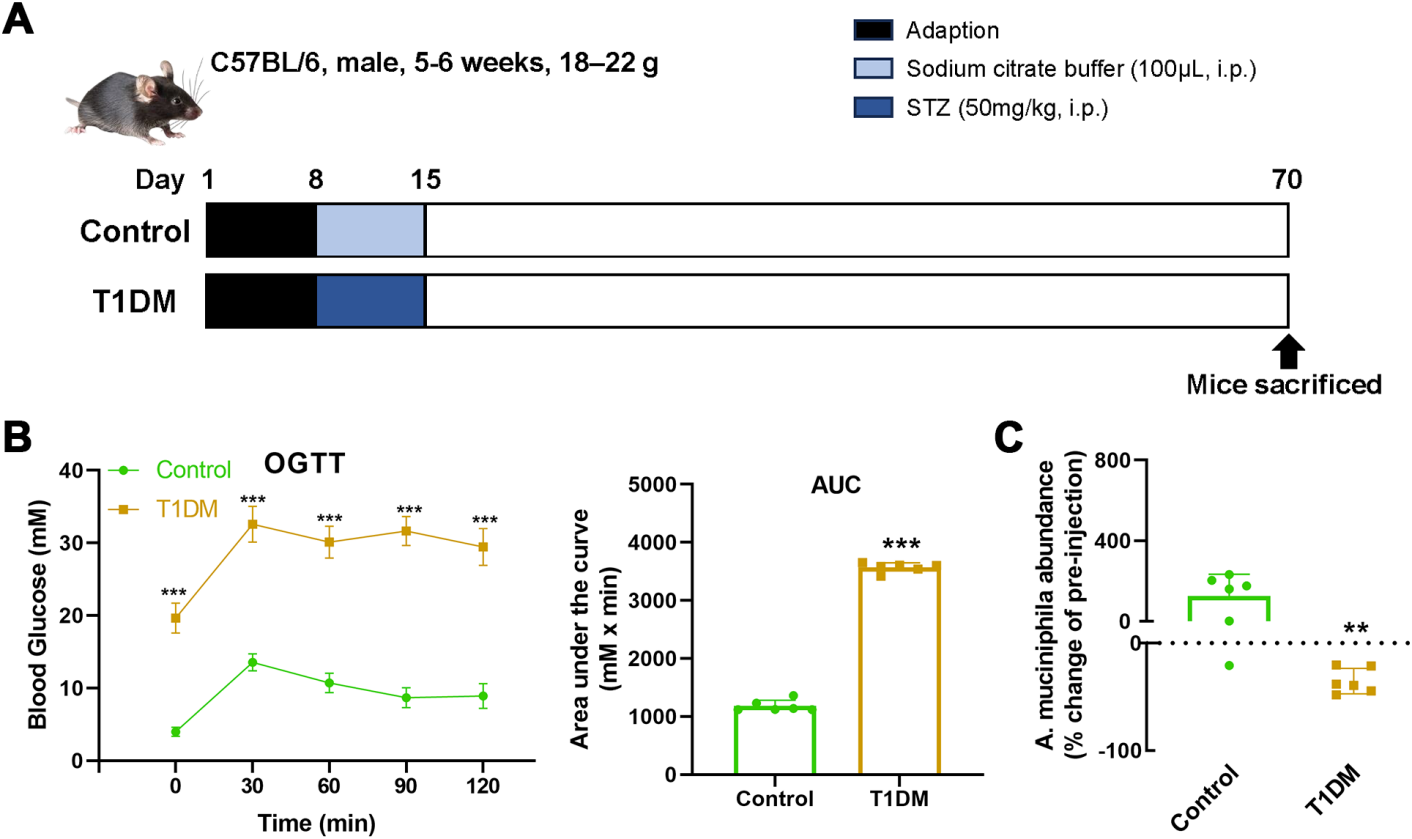
STZ-induced T1DM mice exhibit impaired glucose tolerance and reduced *A. muciniphila* abundance. **(A)** Schematic timeline of the experimental protocol. Male C57BL/6 mice (5–6 weeks old, 18–22 g) were adapted for one week, followed by intraperitoneal injection of sodium citrate buffer (control) or STZ (50 mg/kg/day) for 5 consecutive days to T1DM. Mice were sacrificed at day 70. **(B)** OGTT and AUC analysis in control and T1DM mice. Data are shown as blood glucose levels at indicated time points. **(C)** Relative abundance of *A.muciniphila* in fecal samples at week 8 post-STZ modeling (day 70), expressed as percentage change compared to pre-injection baseline (day 8). Data are presented as mean ± SEM (n = 6 per group). Statistical significance was determined by unpaired Student’s *t*-test. **p < 0.01, ***p < 0.001.

To establish a murine model of T1DM, male C57BL/6 mice (5–6 weeks old) were intraperitoneally injected with STZ (50 mg/kg/day) for five consecutive days following one week of adaptation (**Figure 1A**). Compared to control mice, STZ-induced T1DM mice exhibited marked hyperglycemia, as evidenced by significantly elevated blood glucose levels during the OGTT and increased AUC values (**Figure 1B**). Next, the abundance of *A. muciniphila* in fecal samples collected from mice 10 weeks after modeling was assessed. The result revealed a significant reduction in *A. muciniphila* levels in the T1DM group compared to controls (**Figure 1C**), supporting that STZ-induced diabetes is associated with gut microbiota alterations, particularly a loss of *A. muciniphila*.

### AmEVs improve glucose homeostasis in T1DM mice

**Figure 2:**
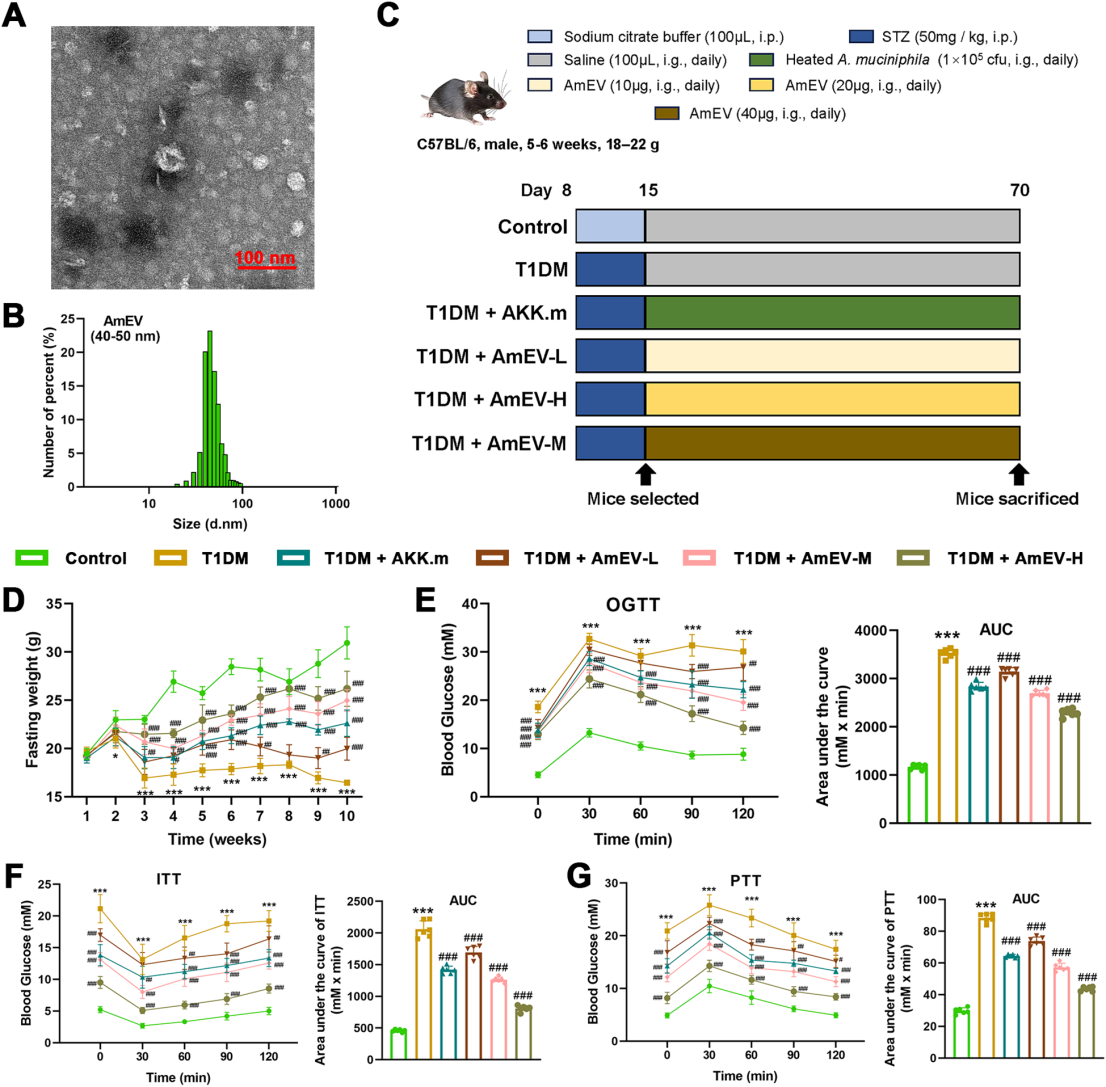
AmEVs improve glucose metabolism and attenuate weight loss in STZ-induced T1DM mice. **(A)** TEM image showing the spherical morphology of AmEVs. Scale bar: 100 nm. **(B)** Size distribution of AmEVs analyzed by NTA, indicating a predominant diameter of 40-50 nm. **(C)** Experimental timeline and treatment groups. **(D)** Body weight changes recorded weekly throughout the treatment period. **(E)** OGTT and corresponding AUC. **(F)** ITT and AUC analysis. **(G)** PTT and AUC analysis. Data are presented as mean ± SEM (n = 6 per group). Statistical significance was determined by one-way ANOVA with Tukey’s *post hoc* test. *p < 0.05, ***p < 0.001 vs. Control group; ##p < 0.01, ###p < 0.001 vs. T1DM group.

To evaluate the therapeutic efficacy of AmEVs, we first confirmed their structural characteristics. TEM revealed that AmEVs displayed typical spherical morphology with a diameter of approximately 40–50 nm (**Figure 2A**), which was further validated by NTA (**Figure 2B**).

T1DM mice were then treated with daily oral gavage of heat-inactivated *A. muciniphila* (10^5^ CFU) or AmEVs at low (10 μg), medium (20 μg), or high (40 μg) doses for 8 weeks (**Figure 2C**). During the treatment period, T1DM mice exhibited a significant reduction in body weight compared to healthy controls, while AmEV administration dose-dependently mitigated weight loss (**Figure 2D**). In addition, AmEVs markedly improved glucose homeostasis. OGTT revealed reduced blood glucose levels and significantly lower AUC values in AmEV-treated groups compared to T1DM mice (**Figure 2E**). Similarly, AmEVs enhanced insulin sensitivity, as evidenced by improved responses in ITT (**Figure 2F**), and restored hepatic gluconeogenesis in PTT (**Figure 2G**). Notably, AmEVs outperformed heat-inactivated *A. muciniphila* in all metabolic assessments (**Figures 2E–G**). These results suggest that AmEVs exert systemic metabolic benefits in T1DM mice, laying the foundation for further investigation into their effects on pancreatic islet preservation.

### AmEVs preserve pancreatic islet structure and insulin secretory function in T1DM mice

**Figure 3:**
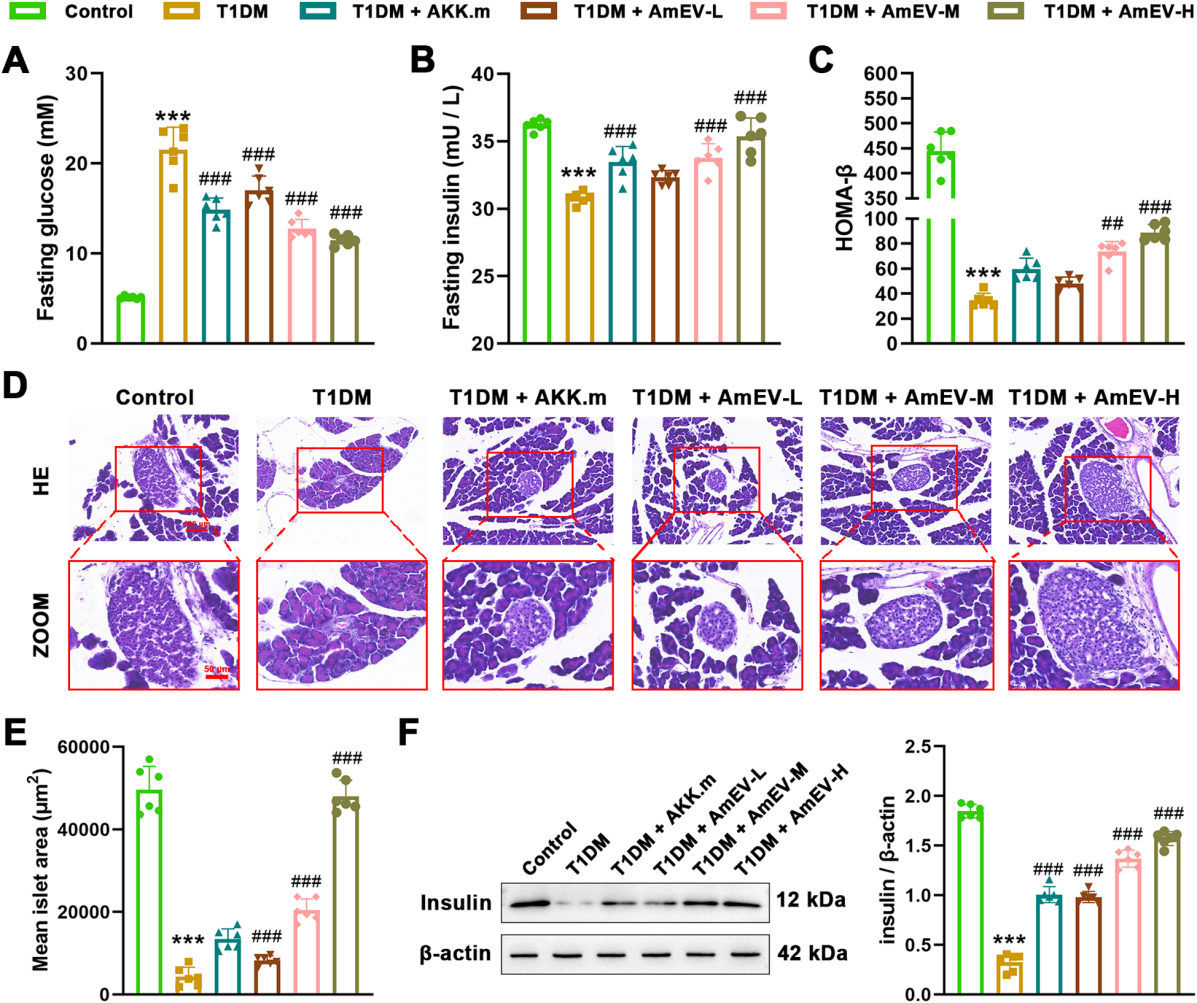
AmEVs preserve pancreatic islet morphology and insulin secretory function in STZ-induced T1DM mice. **(A–C)** FBG, FINS levels, and HOMA-β index in each group. **(D)** Representative H&E-stained pancreatic sections showing islet morphology at low and high magnification. Scale bar = 100 and 50 μm **(E)** Quantification of mean islet area based on ImageJ analysis of H&E sections. **(F)** Western blot analysis of insulin expression in pancreatic tissue and quantification relative to β-actin. Data are presented as mean ± SEM (n = 6 per group). Statistical analysis was performed using one-way ANOVA followed by Tukey’s *post hoc* test. ***p < 0.001 vs. Control group; ##p < 0.01, ###p < 0.001 vs. T1DM group.

To further investigate the protective effects of AmEVs on pancreatic endocrine function, FBG and FINS levels were analyzed. Compared to the T1DM group, both heat-inactivated *A. muciniphila* (AKK.m) and AmEV treatments significantly reduced FBG, with AmEVs showing a more pronounced and dose-dependent effect (**Figure 3A**). Similarly, FINS levels were partially restored in the AKK.m group and further elevated in mice receiving medium and high doses of AmEVs (**Figure 3B**). Notably, the HOMA-β index, which reflects β-cell functional capacity, was not significantly improved in the AKK.m group relative to the T1DM group. In comparison, medium- and high-dose AmEV treatment markedly increased HOMA-β values, indicating an effective restoration of β-cell function (**Figure 3C**). Histological analysis of pancreatic sections revealed that T1DM mice exhibited disrupted islet architecture and significant islet atrophy. In contrast, AmEV-treated mice showed preserved islet morphology and enlarged islet area relative to the T1DM group (**Figure 3D**). Quantification of islet area confirmed significant improvements in all treatment groups, with the high-dose AmEVs group showing near-complete restoration to control levels (**Figure 3E**). To further validate these findings, insulin protein expression in pancreatic tissue was evaluated by western blot. As shown in **Figures 3F**, insulin levels were markedly reduced in the T1DM group, whereas AmEV treatment restored insulin expression in a dose-dependent manner. Notably, medium and high doses of AmEVs exhibited stronger protective effects on islet structure and insulin production compared to heat-inactivated *A. muciniphila*. These results indicate that AmEVs not only ameliorate hyperglycemia but also preserve pancreatic islet integrity and enhance β-cell secretory function in T1DM mice.

### AmEVs improve hepatic lipid metabolism in T1DM mice

**Figure 4:**
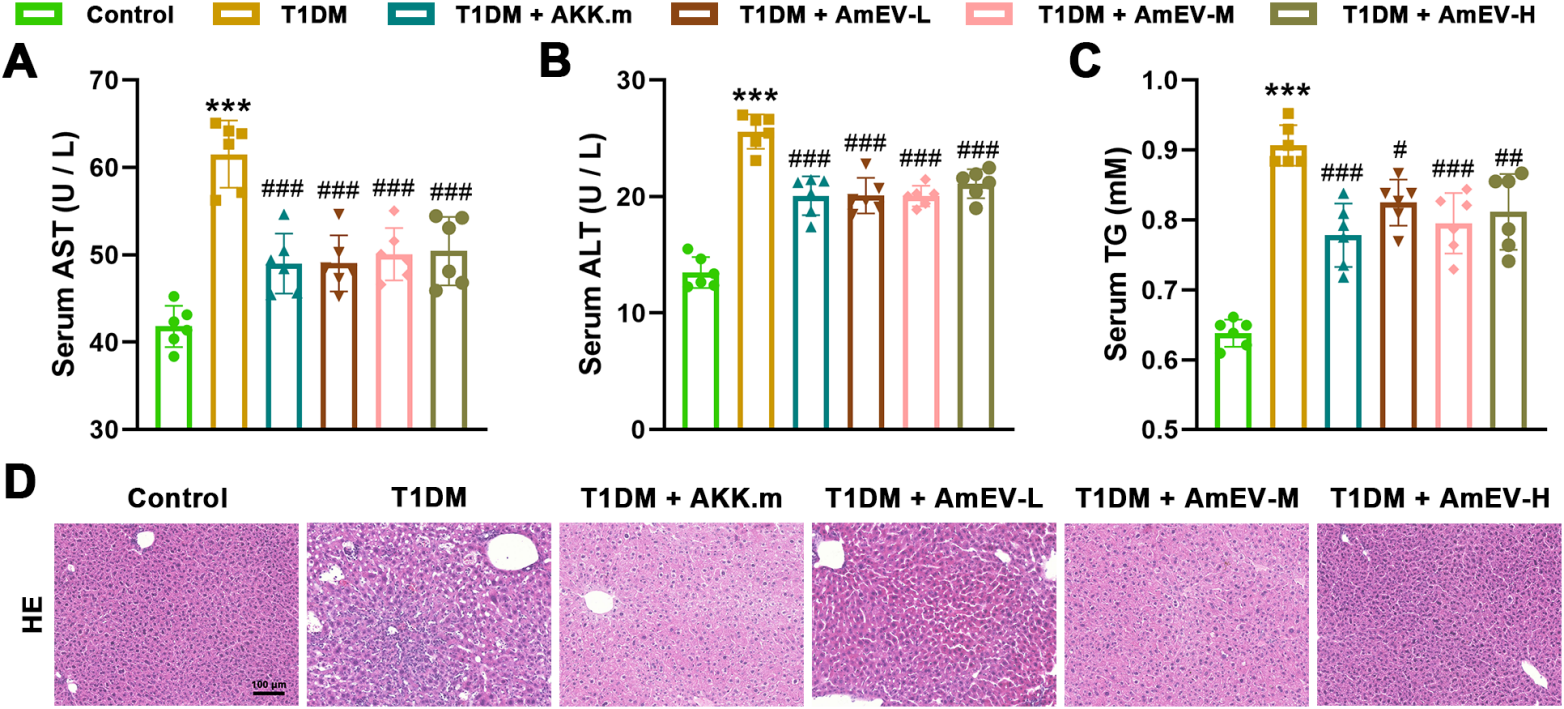
AmEVs improve liver function and lipid metabolism in STZ-induced T1DM mice. **(A–C)** Serum levels of AST, ALT, and TG in each group. **(D)** Representative H&E staining of liver sections showing hepatic architecture and morphology. Scale bar = 100 μm. Data are expressed as mean ± SEM (n = 6 per group). Statistical analysis was performed using one-way ANOVA followed by Tukey’s *post hoc* test. ***p < 0.001 vs. Control group; #p < 0.05, ##p < 0.01, ###p < 0.001 vs. T1DM group.

To evaluate the effects of AmEVs on liver function and lipid metabolism, serum levels of AST, ALT, and TG were measured. As expected, T1DM mice exhibited significantly elevated levels of AST, ALT, and TG compared to the control group, indicating hepatic injury and lipid metabolic disorder (**Figures 4A–C**). Treatment with heat-inactivated *A. muciniphila* and all three doses of AmEVs led to a reduction in these serum markers. Although the overall trend showed improvement across all treatment groups, the differences among the AKK.m and AmEV-treated groups were minimal. H&E staining revealed that T1DM mice displayed marked hepatic structural disruption, including hepatocyte ballooning and cytoplasmic vacuolation. These pathological features were alleviated to a comparable extent in the AKK.m and AmEV groups, with improved hepatocyte morphology and more organized tissue architecture (**Figure 4D**). Collectively, these results indicate that both heat-inactivated A. muciniphila and AmEVs exert protective effects on liver injury and glucolipid metabolic disturbances in T1DM mice, though no significant differences were observed among treatment groups.

### AmEVs attenuate oxidative stress and inflammation in T1DM mice

Oxidative stress and chronic inflammation are key contributors to pancreatic damage in T1DM. To determine whether AmEVs exert antioxidant and anti-inflammatory effects, we measured oxidative stress markers and pro-inflammatory cytokines in both plasma and pancreatic tissues.

**Figure 5:**
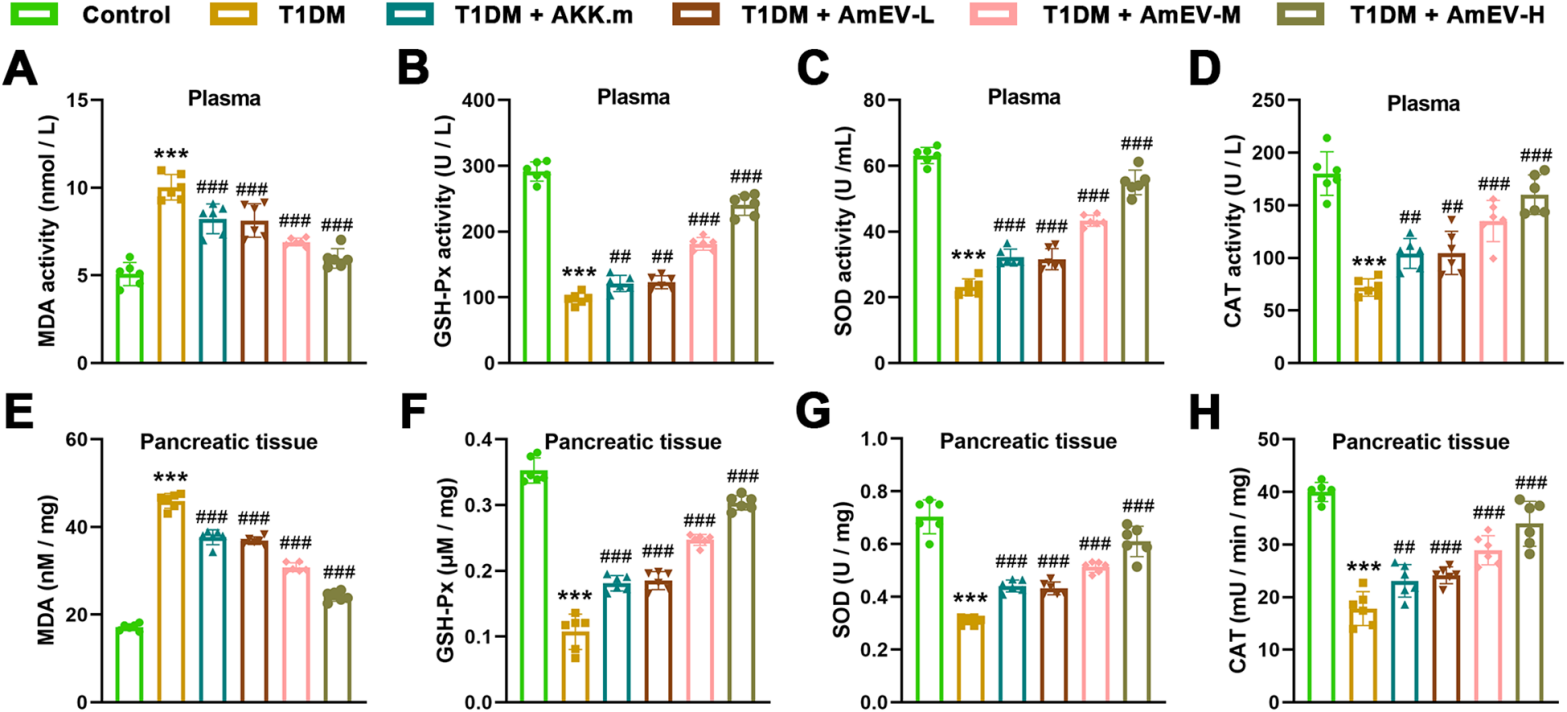
AmEVs reduce oxidative stress in plasma and pancreatic tissues of T1DM mice. **(A–D)** Plasma levels of MDA, GSH-Px, SOD, and CAT in each group. **(E-H)** Corresponding oxidative stress markers measured in pancreatic tissues. Data are expressed as mean ± SEM (n = 6 per group). Statistical analysis was performed using one-way ANOVA followed by Tukey’s *post hoc* test. ***p < 0.001 vs. Control group; ##p < 0.01, ###p < 0.001 vs. T1DM group.

To assess whether AmEVs alleviate oxidative stress in diabetic mice, we measured MDA content and antioxidant enzyme activities in both plasma and pancreatic tissues. In plasma (**Figures 5A–D**), STZ-induced T1DM mice exhibited significantly increased MDA levels and decreased activities of GSH-Px, SOD, and CAT compared to controls. Treatment with heat-inactivated A. muciniphila and AmEVs significantly reduced MDA levels, with the medium and high doses of AmEVs showing greater reductions. All treatment groups also showed significant improvements in GSH-Px, SOD, and CAT activities relative to T1DM. Among them, the AmEV-H group displayed the most robust elevation in antioxidant enzyme activity. A similar trend was observed in pancreatic tissue (**Figures 5E–H**). T1DM mice showed elevated MDA levels and reduced GSH-Px, SOD, and CAT activities. All treatments significantly reversed these alterations. Notably, AmEV-M and AmEV-H groups restored antioxidant enzyme levels close to those of the control group, while the AKK.m group showed moderate improvement.

**Figure 6:**
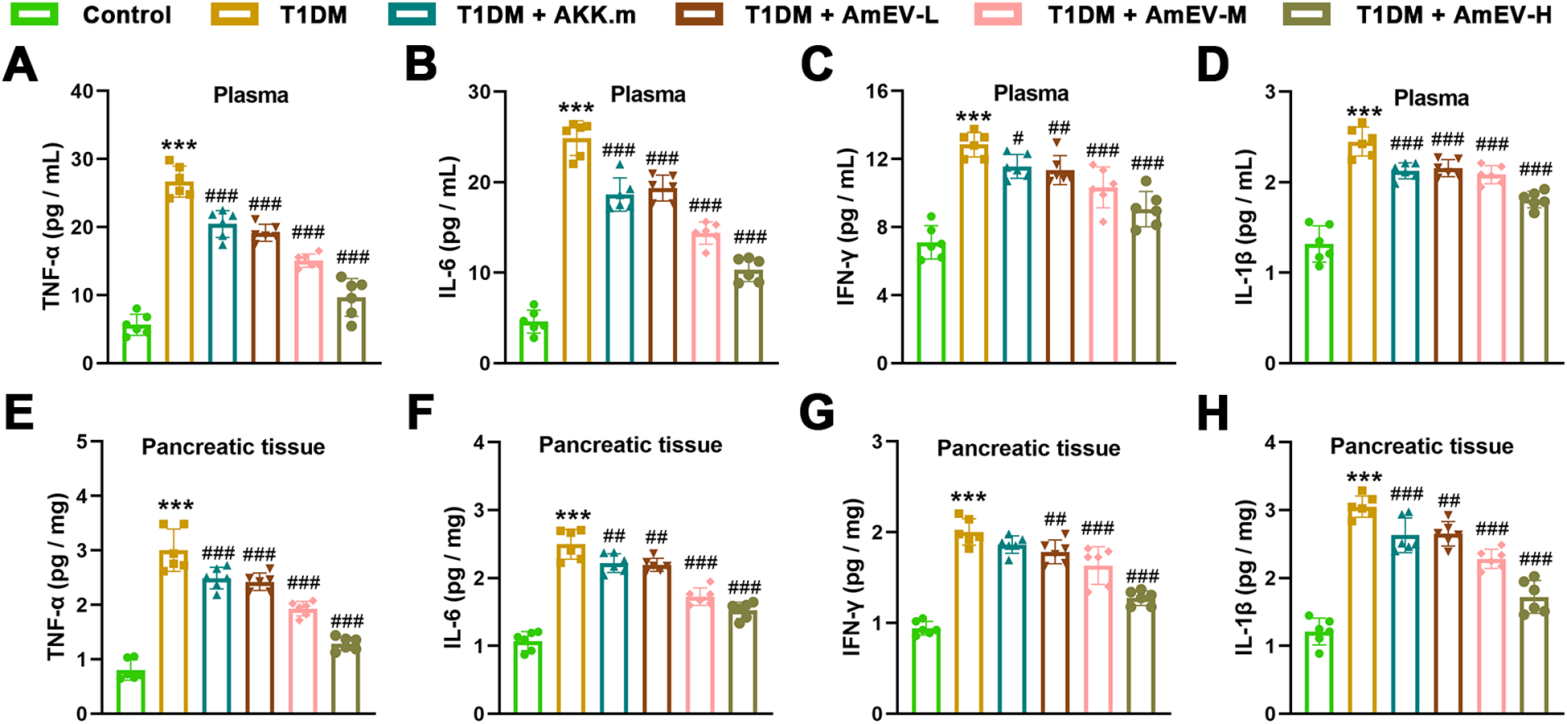
AmEVs suppress inflammatory cytokine production in plasma and pancreas of T1DM mice. **(A–D)** Plasma levels of TNF-α, IL-6, IFN-γ, and IL-1β in each group. **(E–H)** Cytokine concentrations in pancreatic tissues. Data are expressed as mean ± SEM (n = 6 per group). Statistical analysis was performed using one-way ANOVA followed by Tukey’s *post hoc* test. ***p < 0.001 vs. Control group; #p < 0.05, ##p < 0.01, ###p < 0.001 vs. T1DM group.

To investigate inflammatory status, levels of pro-inflammatory cytokines were quantified in plasma (**Figures 6A–D**) and pancreatic tissues (**Figures 6E–H**). Compared to controls, T1DM mice had significantly elevated TNF-α, IL-6, IFN-γ, and IL-1β levels. Both AKK.m and AmEV treatment groups significantly reduced these cytokine levels in plasma and pancreas. AmEV-M and AmEV-H groups showed the greatest reduction in most cytokines, particularly IL-6 and IL-1β, which were significantly lower than those in the AKK.m and AmEV-L groups.

These results collectively demonstrate that both AKK.m and AmEVs mitigate oxidative stress and inflammation in T1DM mice, with AmEVs showing a dose-dependent trend of improved efficacy.

### AmEVs promote pancreatic immune tolerance in T1DM mice

**Figure 7:**
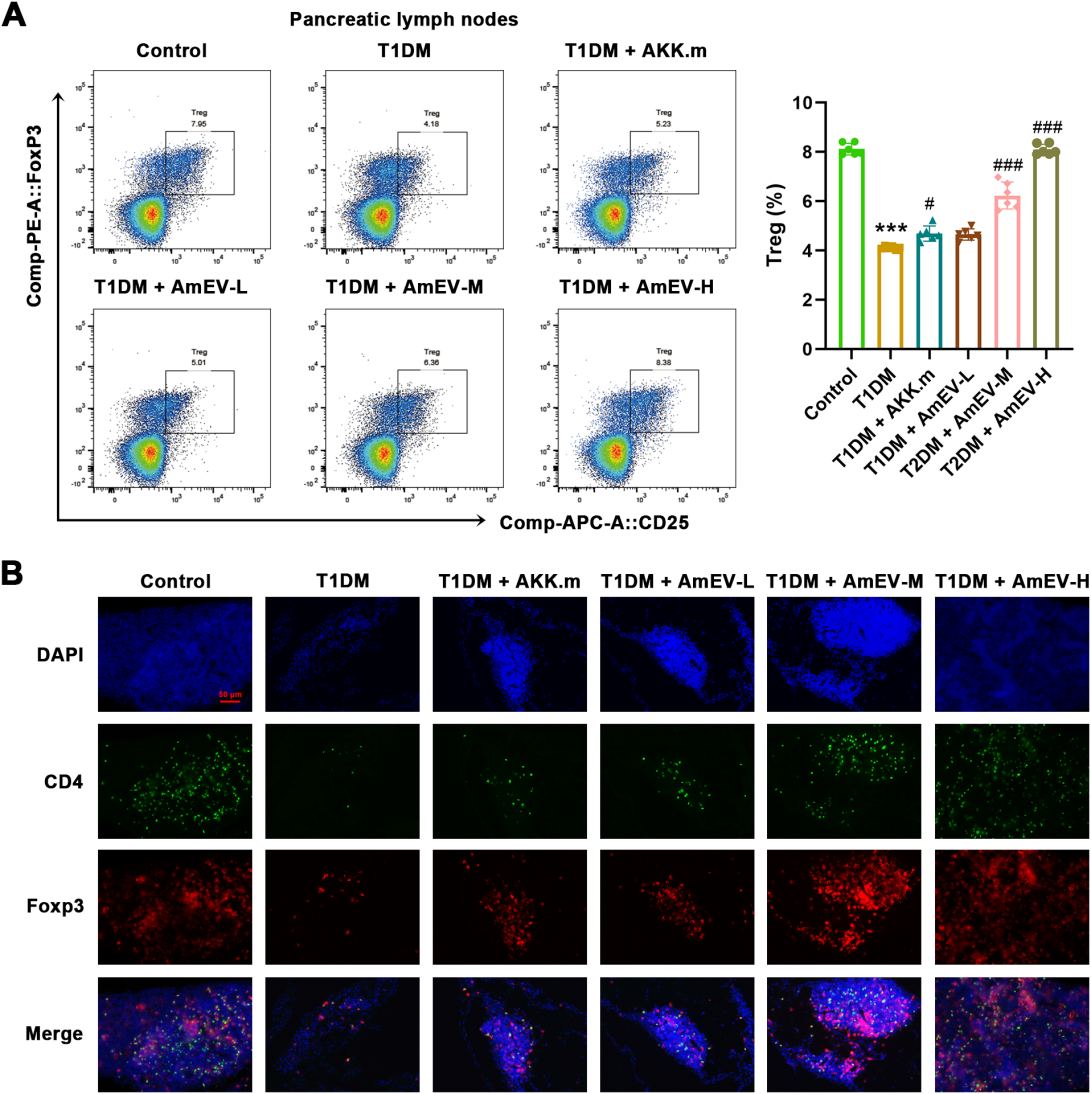
AmEVs restore Treg populations in pancreatic lymph nodes and pancreatic tissues of T1DM mice. **(A)** Flow cytometric analysis of CD4^+^CD25^+^Foxp3^+^ Tregs in pancreatic draining lymph nodes. Representative dot plots (left) and quantification of Treg frequency (right). **(B)** Immunofluorescence staining of pancreatic sections. CD4 (green), Foxp3 (red), nuclei counterstained with DAPI (blue). Merged images show co-localization of CD4^+^Foxp3^+^ Tregs. Scale bar = 100 μm. Data are expressed as mean ± SEM (n = 6 per group). Statistical analysis was performed using one-way ANOVA followed by Tukey’s *post hoc* test. ***p < 0.001 vs. Control group; #p < 0.05, ###p < 0.001 vs. T1DM group.

To explore whether AmEVs modulate local immune tolerance, we examined the frequency of Tregs in pancreatic lymph nodes and their localization in pancreatic tissue. Flow cytometry analysis showed that the proportion of CD4^+^CD25^+^Foxp3^+^ Tregs was significantly reduced in T1DM mice compared to controls (**Figure 7A**). Treatment with heat-inactivated *A. muciniphila* partially restored Treg frequency, while AmEVs induced a dose-dependent increase in Tregs, with the AmEV-H group showing the highest percentage, approaching control levels. Consistently, immunofluorescence staining of pancreatic sections revealed a marked decrease in Foxp3^+^CD4^+^ Tregs in T1DM mice (**Figure 7B**). Both AKK.m and AmEV treatments promoted the presence of Foxp3^+^CD4^+^ Tregs in pancreatic tissue, with stronger Foxp3 and CD4 co-localization observed in AmEV-M and AmEV-H groups. These findings indicate that AmEVs enhance Treg-associated pancreatic immune regulation, potentially through the expansion and local enrichment of Tregs.

### Treg depletion attenuates the protective effects of AmEVs

**Figure 8:**
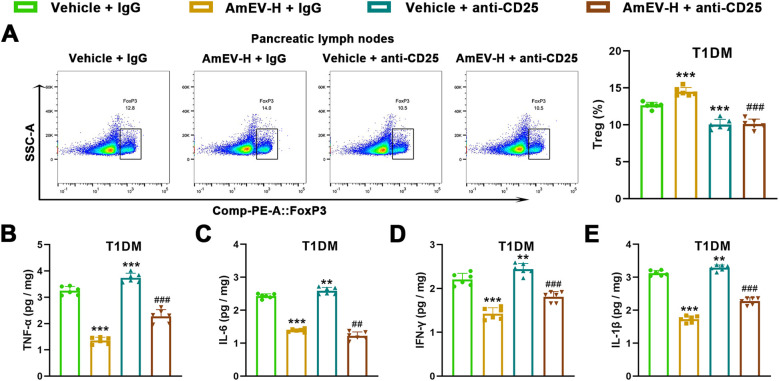
Anti-CD25–mediated Treg depletion reverses AmEV–induced immunoregulation and anti-inflammatory effects in STZ-induced T1DM mice. **(A)** Flow cytometric analysis of CD4^+^Foxp3^+^ Tregs in pancreatic draining lymph nodes. Representative gating plots (left) and quantification of Treg frequency (right) from the indicated groups. **(B–E)** Concentrations of TNF-α, IL-6, IFN-γ, and IL-1β in pancreatic tissue homogenates. Data are presented as mean ± SEM (n = 6 per group). Statistical analysis was performed using one-way ANOVA followed by Tukey’s *post hoc* test. ***p < 0.001 vs. Vehicle + IgG group; ##p < 0.01, ###p < 0.001 vs. AmEV-H + IgG group.

To determine whether Tregs contribute functionally to the immunomodulatory effects of AmEVs, a Treg depletion experiment was performed using an anti-CD25 antibody. Flow cytometric analysis confirmed that anti-CD25 treatment markedly reduced the proportion of CD4^+^Foxp3^+^ Tregs in pancreatic draining lymph nodes (**Figure 8A**). Importantly, depletion of CD25^+^ T cells significantly attenuated the anti-inflammatory effects of AmEV-H treatment. Compared with the T1DM + AmEV-H group, mice treated with an anti-CD25 antibody exhibited increased levels of pro-inflammatory cytokines, including TNF-α, IL-6, IFN-γ, and IL-1β (**Figures 8B–E**).

**Figure 9:**
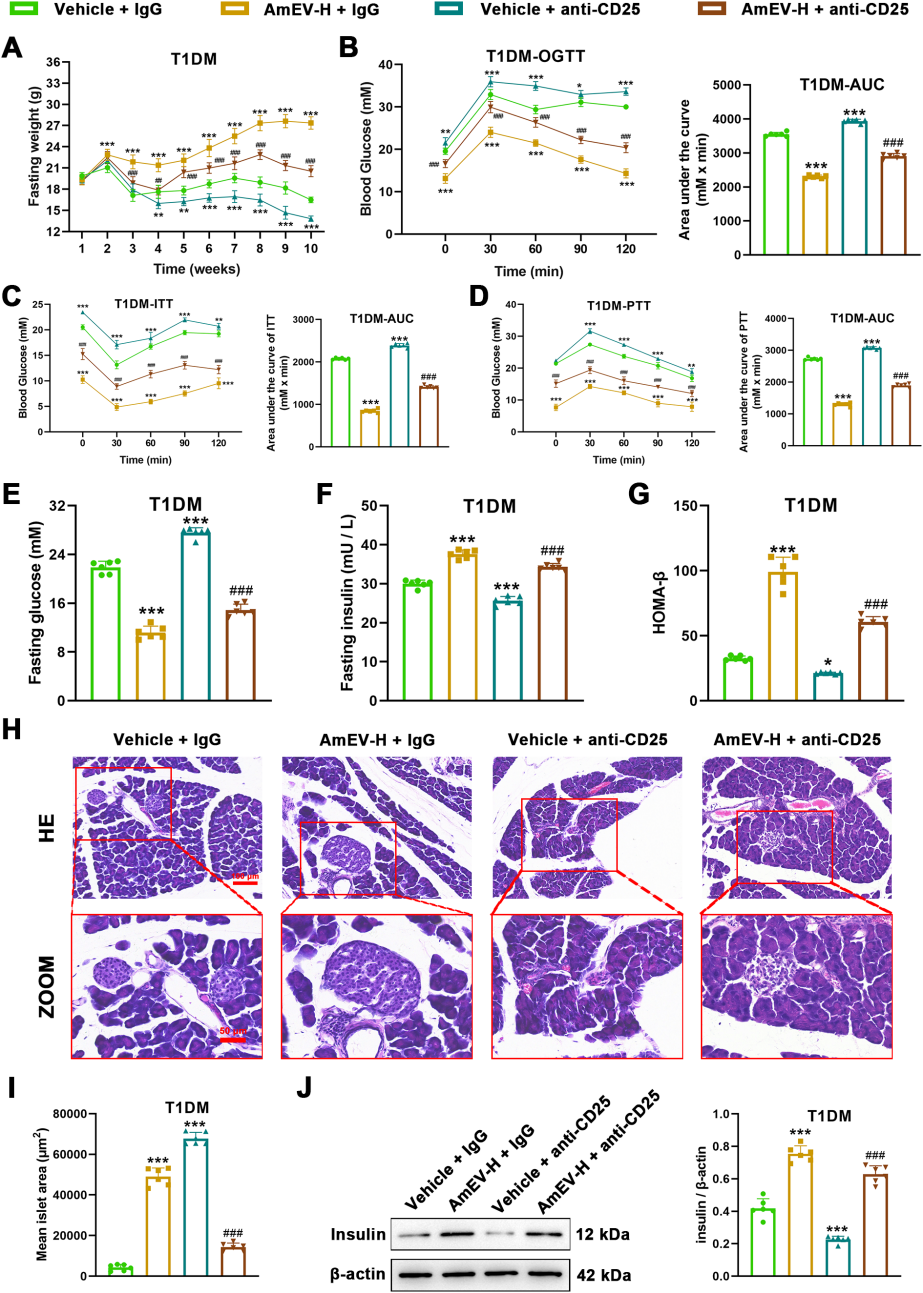
Anti-CD25–mediated Treg depletion attenuates the metabolic and islet-protective effects of AmEVs in STZ-induced T1DM mice. **(A)** Body weight changes recorded weekly throughout the treatment period in the four groups (Vehicle + IgG, AmEV-H + IgG, Vehicle + anti-CD25, and AmEV-H + anti-CD25). **(B)** OGTT and corresponding AUC. **(C)** ITT and AUC analysis. **(D)** PTT and AUC analysis. **(E-G)** FBG, FINS levels, and HOMA-β index in each group. **(H)** Representative H&E-stained pancreatic sections showing islet morphology at low and high magnification. Scale bar = 100 and 50 μm **(I)** Quantification of mean islet area based on ImageJ analysis of H&E sections. **(J)** Western blot analysis of insulin expression in pancreatic tissue and quantification relative to β-actin. Data are presented as mean ± SEM (n = 6 per group). Time-course data (body weight, OGTT, ITT, and PTT curves) were analyzed using two-way repeated-measures ANOVA followed by Tukey’s *post hoc* test. Single time-point comparisons were analyzed using one-way ANOVA with Tukey’s multiple comparisons test. ***p < 0.001 vs. Vehicle + IgG group; ##p < 0.01, ###p < 0.001 vs. AmEV-H + IgG group.

Moreover, metabolic parameters were also analyzed following Treg depletion. Compared with the T1DM + AmEV-H group, anti-CD25 treatment significantly weakened the beneficial effects of AmEV-H on body weight, glucose tolerance, and insulin secretion (**Figures 9A–J**). Specifically, mice treated with an anti-CD25 antibody exhibited elevated fasting blood glucose, impaired OGTT responses, and reduced serum insulin levels compared with mice treated with AmEV-H alone. These results indicate that the metabolic improvements induced by AmEVs are at least partially mediated through Treg-dependent immunoregulation.

Final, we further assessed gut barrier–related parameters and systemic endotoxemia to explore potential upstream mechanisms contributing to the immunomodulatory effects of AmEVs. AmEV-H treatment significantly reduced circulating LPS levels and partially restored the expression of intestinal tight junction proteins compared with the T1DM group (**Supplementary Figure 2**). Although these findings were exploratory, they suggest that AmEVs may alleviate diabetes-associated gut barrier dysfunction and metabolic endotoxemia, thereby contributing to the improvement of the inflammatory immune milieu.

## Discussion

The therapeutic use of gut-derived EVs is emerging as a next-generation strategy distinct from live bacterial supplementation. In this study, we demonstrate that AmEVs exert significant protective effects in a mouse model of T1DM. AmEV treatment ameliorated hyperglycemia, preserved pancreatic islet structure and insulin secretion, improved systemic metabolic parameters including hepatic and lipid metabolic profiles, reduced oxidative stress and inflammation, and promoted Treg-associated immunoregulation through expansion of regulatory T cell populations. Notably, the immunological and pancreatic protective effects of AmEVs were more pronounced than those observed with heat-inactivated *A. muciniphila*, particularly at medium and high AmEV doses.

Our analysis revealed a marked reduction in fecal *A. muciniphila* abundance in STZ-induced T1DM mice, which is consistent with previous reports in both metabolic and autoimmune contexts. Studies in obese or insulin-resistant humans and mice have repeatedly shown that *A. muciniphila* levels are inversely correlated with metabolic impairment (30, 31). For example, in NOD mice, *A. muciniphila* is almost absent in colonies with high diabetes incidence, and its oral supplementation reshapes gut microbiota, reduces endotoxin levels, and delays disease onset by enhancing Treg responses (29). Similarly, in metformin-treated obese mice, the expansion of *A. muciniphila* has been shown to improve glucose homeostasis, accompanied by increased mucin production and intestinal Treg accumulation (32). Evidence from other disease contexts also suggests that the loss of certain commensals is not merely correlative but may reflect a biologically meaningful disruption in host-microbe interactions. For instance, the abundance of *Faecalibacterium prausnitzii* is significantly reduced in patients with Crohn’s disease (33). Its culture supernatant exhibits anti-inflammatory properties, such as suppression of NF-κB activation, and alleviates colitis severity in mouse models (33). In allergic and atopic disease models, *Bifidobacterium breve* and *Lactobacillus reuteri* are frequently diminished. Restoration of these species promotes immunoregulatory function (34). These findings support the idea that loss of commensals with immunomodulatory function may contribute directly to disease progression. Our observed reduction of *A. muciniphila* in the T1DM model fits this framework, and highlights its potential as a therapeutic target.

A key distinction of this study lies in the use of AmEVs, rather than inactivated bacteria. Although previous work has demonstrated that pasteurized *A. muciniphila* can improve glucose tolerance and reduce adiposity in NOD mice (30), its efficacy in autoimmune settings such as T1DM remains limited and inconsistent. In our model, we observed that heat-inactivated *A. muciniphila* partially improved glucose metabolism and inflammation but did not restore β-cell function or immune tolerance to the same extent as AmEVs. This suggests that EVs may offer functional advantages beyond what can be achieved by the inactivated bacterium. A recent study demonstrated that AmEVs has a better effect compared with pasteurized *A. muciniphila* on intestinal ischemia-reperfusion-induced postoperative cognitive dysfunction (35). This superiority is also supported by accumulating evidence from other bacterial species. For example, EVs derived from *Lactobacillus johnsonii* or *Bifidobacterium longum* have been shown to elicit stronger immunomodulatory effects than their respective whole-cell counterparts, likely due to their ability to concentrate and deliver specific microbial ligands to host target cells (36, 37). From a therapeutic standpoint, EVs present several advantages. They are more stable and scalable than live bacteria, easier to standardize and store, and carry lower risks of microbial overgrowth, especially in immunocompromised hosts (38). Importantly, their composition can be engineered or fractionated to isolate specific bioactive elements, enabling mechanism-driven therapy development. In our study, the consistent dose-response relationship observed with AmEVs further supports the notion that EVs act as structured, biologically active delivery units, rather than passive by-products.

Oxidative stress and inflammation are not secondary consequences of hyperglycemia, but rather primary contributors to β-cell destruction and immune activation in T1DM. Chronic exposure of pancreatic islets to reactive oxygen species sensitizes β-cells to apoptosis by impairing mitochondrial function, DNA integrity, and insulin gene transcription (39). Concurrently, inflammatory cytokines such as TNF-α, IL-1β, and IFN-γ, released by activated macrophages and T cells, induce nitric oxide production and activate NF-κB and STAT1 pathways, accelerating β-cell death and antigen presentation (40, 41). These processes form a self-perpetuating loop, in which metabolic and immune stressors amplify each other, breaking tolerance and promoting autoimmunity (42). Therapeutic strategies that target this oxidative-inflammatory axis have shown broad efficacy. For example, glucagon-like peptide 1 effectively alleviated inflammation and oxidative stress induced by hypoglycemia and hyperglycemia, which was proven to be effective in TIDM management (43). Similarly, agents that inhibit NF-κB or JNK signaling can dampen inflammatory cascades and delay T1DM onset mice (44). In this context, our findings show that AmEVs significantly reduce both systemic and pancreatic oxidative stress and inflammation, suggesting that these vesicles act through converging metabolic and immune regulatory pathways. By attenuating redox imbalance and cytokine signaling, AmEVs may disrupt the positive-feedback loop between inflammation and β-cell stress, thus preserving islet integrity and immune quiescence. Compared with heat-inactivated *A. muciniphila*, AmEVs showed more consistent reductions across tissues and cytokine types, reinforcing the concept that bacterial EVs may more effectively deliver immunomodulatory signals to distal sites.

Recent studies have also highlighted the role of gut barrier dysfunction and endotoxemia in metabolic and immune dysregulation associated with diabetes (45, 46). Increased circulating LPS can promote systemic inflammation and exacerbate β-cell stress through activation of innate immune signaling pathways (47). In the present study, AmEV treatment was associated with reduced circulating LPS levels and improved expression of intestinal tight junction proteins. Although these observations were exploratory, they suggest that AmEVs may partially improve immune homeostasis by alleviating metabolic endotoxemia and restoring gut barrier integrity, which may in turn contribute to a more favorable immunoregulatory environment.

A hallmark of T1DM pathogenesis is the breakdown of peripheral and local immune tolerance, leading to autoreactive T cell infiltration and progressive β-cell destruction. Tregs are central regulators of immune homeostasis and can suppress multiple immune compartments involved in T1DM pathogenesis (48). Defects in Treg number, stability, or function have been implicated in both murine and human T1DM, and restoring Treg-mediated suppression has emerged as a key therapeutic goal (49). In our study, AmEVs significantly increased the proportion of Tregs in pancreatic draining lymph nodes and enhanced Tregs infiltration into pancreatic islets. These effects were more robust than those achieved by heat-inactivated *A. muciniphila*, suggesting that vesicle-derived components may more effectively engage immune-regulatory pathways. Several potential mechanisms may underlie this effect. First, oxidative stress is known to destabilize Tregs (50), while antioxidant interventions can preserve Treg identity and function. The potent antioxidant effects of AmEVs may therefore indirectly support Treg lineage stability. Second, TLR2 stimulation in a tolerogenic context has been reported to promote Treg induction through dendritic cell modulation, and *A. muciniphila* membrane proteins including Amuc_1100 are known TLR2 ligands (30). Given that bacterial extracellular vesicles are enriched in membrane-associated components, it is plausible that TLR2-related signaling may contribute to the immunomodulatory effects of AmEVs. However, this possibility was not directly tested in the present study and therefore should be interpreted cautiously. Future studies using pharmacologic inhibition or genetic blockade of TLR2/4 signaling will be necessary to determine whether these pathways are required for AmEV-mediated Treg regulation. This is consistent with prior work showing that gut-derived microbial signals can influence systemic immune balance. For example, polysaccharide A from *Bacteroides fragilis* promotes Treg differentiation via TLR2-dependent pathways, reducing inflammation in colitis and EAE models (51). Similarly, *A. muciniphila* was shown to enhance Treg numbers and suppress autoreactive T cell responses in NOD mice, although the responsible mediators were not fully defined (29). Our data extend these findings by suggesting that extracellular vesicles may represent one mediator capable of modulating immune regulation in T1DM. Taken together, the enhancement of Treg responses may represent a key immunological mechanism through which AmEVs promote islet preservation and long-term disease attenuation.

In addition to their direct immunosuppressive activity, Tregs are known to influence multiple immune compartments involved in T1DM pathogenesis. For example, Tregs can suppress effector T-cell subsets such as Th1 and Th17 cells (52), both of which contribute to β-cell destruction through proinflammatory cytokine production (52, 53). Tregs also regulate dendritic cell maturation and antigen-presenting function through cytokines such as IL-10 and TGF-β, thereby shaping the inflammatory microenvironment (54). Therefore, the expansion of Tregs observed following AmEV treatment may indirectly contribute to broader immune regulation beyond the Treg compartment itself. Future studies examining additional immune populations will help further clarify the immune network underlying AmEV-mediated protection.

Importantly, our anti-CD25 depletion experiment provides functional evidence supporting the role of Tregs in mediating the protective effects of AmEVs. Depletion of CD25^+^ T cells markedly attenuated the anti-inflammatory and metabolic benefits induced by AmEV-H treatment, including worsening glycemic control, reduced insulin levels, and increased pro-inflammatory cytokine production. These findings suggest that the beneficial effects of AmEVs are at least partially dependent on Treg-mediated immunoregulation, providing functional support for the role of Tregs in mediating the protective effects of AmEVs in this model. Rather than representing a purely correlative association, the expansion of Tregs appears to functionally contribute to the restoration of immune balance and the protection of pancreatic islets in this model.

From a translational perspective, the timing of intervention is an important consideration. In the present study, AmEV administration was initiated shortly after STZ induction in order to evaluate whether early immunometabolic modulation could mitigate the progression of β-cell injury and inflammatory responses. Therefore, the observed benefits may reflect a combination of preventive and early therapeutic effects. Future studies employing delayed treatment paradigms will be necessary to determine whether AmEVs retain efficacy in established or late-stage diabetes. In addition, several factors should be considered when evaluating the clinical potential of bacterial extracellular vesicles. These include their stability within the gastrointestinal environment, bioavailability following oral administration, scalability of vesicle production, and long-term safety. Although bacterial EVs have shown promising safety profiles in preclinical studies, further investigation will be required to establish optimal dosing strategies and evaluate their therapeutic potential in chronic disease settings.

While our findings provide strong evidence that AmEVs exert multifaceted protective effects in T1DM, several limitations warrant consideration. First, this study focused on a preventive/interventional model in STZ-induced T1DM, which mimics some but not all aspects of human autoimmune diabetes. Second, although we observed clear immunomodulatory effects, the precise molecular components within AmEVs responsible for these actions remain to be identified, and the potential involvement of pathways such as TLR2-mediated signaling requires further mechanistic investigation. In addition, the mechanisms by which AmEVs promote Treg stabilization and potentially influence antigen-presenting cell function were not directly examined in this study and therefore remain to be clarified. Third, long-term safety and efficacy in chronic settings and in combination with other therapies require further investigation. Future studies integrating proteomic and lipidomic profiling of AmEV cargo with targeted pathway validation will help elucidate the molecular basis of these immunomodulatory effects.

In conclusion, our study highlights the potential of AmEVs as a novel immunometabolic therapy for T1DM. By integrating metabolic regulation, oxidative stress mitigation, and Treg-associated immune regulation, AmEVs represent a compelling platform for microbiota-inspired intervention. Future work to deconstruct and refine their bioactive content may pave the way for rational, vesicle-based strategies in autoimmune and metabolic diseases.

## Data Availability

The original contributions presented in the study are included in the article/**Supplementary Material**. Further inquiries can be directed to the corresponding author.

## References

[1] Popoviciu MS Kaka N Sethi Y Patel N Chopra H Cavalu S . Type 1 Diabetes Mellitus and Autoimmune Diseases: A Critical Review of the Association and the Application of Personalized Medicine. J personalized Med. (2023) 13:422. doi: 10.3390/jpm13030422, PMID: 36983604 PMC10056161

[2] Janež A Guja C Mitrakou A Lalic N Tankova T Czupryniak L . Insulin Therapy in Adults with Type 1 Diabetes Mellitus: a Narrative Review. Diabetes therapy: research Treat Educ Diabetes related Disord. (2020) 11:387–409. doi: 10.1007/s13300-019-00743-7, PMID: 31902063 PMC6995794

[3] Tatovic D Dayan CM . Replacing insulin with immunotherapy: Time for a paradigm change in Type 1 diabetes. Diabetic medicine: J Br Diabetic Assoc. (2021) 38:e14696. doi: 10.1111/dme.14696, PMID: 34555209

[4] Lachin JM Genuth S Cleary P Davis MD Nathan DM . Retinopathy and nephropathy in patients with type 1 diabetes four years after a trial of intensive therapy. New Engl J Med. (2000) 342:381–9. doi: 10.1056/nejm200002103420603, PMID: 10666428 PMC2630213

[5] Boldison J Wong FS . Immune and Pancreatic β Cell Interactions in Type 1 Diabetes. Trends Endocrinol metabolism: TEM. (2016) 27:856–67. doi: 10.1016/j.tem.2016.08.007, PMID: 27659143

[6] Jeker LT Bour-Jordan H Bluestone JA . Breakdown in peripheral tolerance in type 1 diabetes in mice and humans. Cold Spring Harbor Perspect Med. (2012) 2:a007807. doi: 10.1101/cshperspect.a007807, PMID: 22393537 PMC3282496

[7] Pipella J Motlagh RA Rampazzo Morelli N Thompson PJ . Autoreactive T Cells and Cytokine Stress Drive β-Cell Senescence Entry and Accumulation in Type 1 Diabetes. Diabetes. (2025) 74(9):1562–76. doi: 10.2337/db24-1122, PMID: 40498653 PMC12365421

[8] Hull CM Peakman M Tree TIM . Regulatory T cell dysfunction in type 1 diabetes: what’s broken and how can we fix it? Diabetologia. (2017) 60:1839–50. doi: 10.1007/s00125-017-4377-1, PMID: 28770318 PMC6448885

[9] Ozgur BA Cinar SA Coskunpinar E Yilmaz A Altunkanat D Deniz G . The role of cytokines and T-bet, GATA3, ROR-γt, and FOXP3 transcription factors of T cell subsets in the natural clinical progression of Type 1 Diabetes. Immunol Res. (2023) 71:451–62. doi: 10.1007/s12026-022-09355-z, PMID: 36595206

[10] Thirawatananond P Brown ME Sachs LK Arnoletti JM Yeh WI Posgai AL . Treg-Specific CD226 Deletion Reduces Diabetes Incidence in NOD Mice by Improving Regulatory T-Cell Stability. Diabetes. (2023) 72:1629–40. doi: 10.2337/db23-0307, PMID: 37625150 PMC10588280

[11] Lamichhane S Kemppainen E Trošt K Siljander H Hyöty H Ilonen J . Circulating metabolites in progression to islet autoimmunity and type 1 diabetes. Diabetologia. (2019) 62:2287–97. doi: 10.1007/s00125-019-04980-0, PMID: 31444528 PMC6861356

[12] Vaarala O Atkinson MA Neu J . The “perfect storm” for type 1 diabetes: the complex interplay between intestinal microbiota, gut permeability, and mucosal immunity. Diabetes. (2008) 57:2555–62. doi: 10.2337/db08-0331, PMID: 18820210 PMC2551660

[13] Macia L Thorburn AN Binge LC Marino E Rogers KE Maslowski KM . Microbial influences on epithelial integrity and immune function as a basis for inflammatory diseases. Immunol Rev. (2012) 245:164–76. doi: 10.1111/j.1600-065X.2011.01080.x, PMID: 22168419

[14] Furusawa Y Obata Y Fukuda S Endo TA Nakato G Takahashi D . Commensal microbe-derived butyrate induces the differentiation of colonic regulatory T cells. Nature. (2013) 504:446–50. doi: 10.1038/nature12721, PMID: 24226770

[15] Ioannou A Berkhout MD Geerlings SY Belzer C . Akkermansia muciniphila: biology, microbial ecology, host interactions and therapeutic potential. Nat Rev Microbiol. (2025) 23:162–77. doi: 10.1038/s41579-024-01106-1, PMID: 39406893

[16] Chen X Li P Liu M Zheng H He Y Chen MX . Gut dysbiosis induces the development of pre-eclampsia through bacterial translocation. Gut. (2020) 69:513–22. doi: 10.1136/gutjnl-2019-319101, PMID: 31900289

[17] Kang EJ Cha MG Kwon GH Han SH Yoon SJ Lee SK . Akkermansia muciniphila improve cognitive dysfunction by regulating BDNF and serotonin pathway in gut-liver-brain axis. Microbiome. (2024) 12:181. doi: 10.1186/s40168-024-01924-8, PMID: 39342324 PMC11438137

[18] Zhang J Ni Y Qian L Fang Q Zheng T Zhang M . Decreased Abundance of Akkermansia muciniphila Leads to the Impairment of Insulin Secretion and Glucose Homeostasis in Lean Type 2 Diabetes. Advanced Sci (Weinheim Baden-Wurttemberg Germany). (2021) 8:e2100536. doi: 10.1002/advs.202100536, PMID: 34085773 PMC8373164

[19] Yoon HS Cho CH Yun MS Jang SJ You HJ Kim JH . Akkermansia muciniphila secretes a glucagon-like peptide-1-inducing protein that improves glucose homeostasis and ameliorates metabolic disease in mice. Nat Microbiol. (2021) 6:563–73. doi: 10.1038/s41564-021-00880-5, PMID: 33820962

[20] Chelakkot C Choi Y Kim DK Park HT Ghim J Kwon Y . Akkermansia muciniphila-derived extracellular vesicles influence gut permeability through the regulation of tight junctions. Exp Mol Med. (2018) 50:e450. doi: 10.1038/emm.2017.282, PMID: 29472701 PMC5903829

[21] Ansaldo E Slayden LC Ching KL Koch MA Wolf NK Plichta DR . Akkermansia muciniphila induces intestinal adaptive immune responses during homeostasis. Sci (New York NY). (2019) 364:1179–84. doi: 10.1126/science.aaw7479, PMID: 31221858 PMC6645389

[22] Sultan S Mottawea W Yeo J Hammami R . Gut Microbiota Extracellular Vesicles as Signaling Molecules Mediating Host-Microbiota Communications. Int J Mol Sci. (2021) 22:13166. doi: 10.3390/ijms222313166, PMID: 34884969 PMC8658398

[23] Díaz-Garrido N Badia J Baldomà L . Microbiota-derived extracellular vesicles in interkingdom communication in the gut. J extracellular vesicles. (2021) 10:e12161. doi: 10.1002/jev2.12161, PMID: 34738337 PMC8568775

[24] Kim JY Kim CW Oh SY Jang S Yetunde OZ Kim BA . Akkermansia muciniphila extracellular vesicles have a protective effect against hypertension. Hypertension research: Off J Japanese Soc Hypertension. (2024) 47:1642–53. doi: 10.1038/s41440-024-01627-5, PMID: 38503939

[25] Zheng T Hao H Liu Q Li J Yao Y Liu Y . Effect of Extracelluar Vesicles Derived from Akkermansia muciniphila on Intestinal Barrier in Colitis Mice. Nutrients. (2023) 15:4722. doi: 10.3390/nu15224722, PMID: 38004116 PMC10674789

[26] Keshavarz Azizi Raftar S Ashrafian F Yadegar A Lari A Moradi HR Shahriary A . The Protective Effects of Live and Pasteurized Akkermansia muciniphila and Its Extracellular Vesicles against HFD/CCl4-Induced Liver Injury. Microbiol spectrum. (2021) 9:e0048421. doi: 10.1128/Spectrum.00484-21, PMID: 34549998 PMC8557882

[27] Keshavarz Aziziraftar S Bahrami R Hashemi D Shahryari A Ramezani A Ashrafian F . The beneficial effects of Akkermansia muciniphila and its derivatives on pulmonary fibrosis. Biomedicine pharmacotherapy = Biomedecine pharmacotherapie. (2024) 180:117571. doi: 10.1016/j.biopha.2024.117571, PMID: 39418965

[28] Greer RL Dong X Moraes AC Zielke RA Fernandes GR Peremyslova E . Akkermansia muciniphila mediates negative effects of IFNγ on glucose metabolism. Nat Commun. (2016) 7:13329. doi: 10.1038/ncomms13329, PMID: 27841267 PMC5114536

[29] Hänninen A Toivonen R Pöysti S Belzer C Plovier H Ouwerkerk JP . Akkermansia muciniphila induces gut microbiota remodelling and controls islet autoimmunity in NOD mice. Gut. (2018) 67:1445–53. doi: 10.1136/gutjnl-2017-314508, PMID: 29269438

[30] Plovier H Everard A Druart C Depommier C Van Hul M Geurts L . A purified membrane protein from Akkermansia muciniphila or the pasteurized bacterium improves metabolism in obese and diabetic mice. Nat Med. (2017) 23:107–13. doi: 10.1038/nm.4236, PMID: 27892954

[31] Everard A Belzer C Geurts L Ouwerkerk JP Druart C Bindels LB . Cross-talk between Akkermansia muciniphila and intestinal epithelium controls diet-induced obesity. Proc Natl Acad Sci United States America. (2013) 110:9066–71. doi: 10.1073/pnas.1219451110, PMID: 23671105 PMC3670398

[32] Shin NR Lee JC Lee HY Kim MS Whon TW Lee MS . An increase in the Akkermansia spp. population induced by metformin treatment improves glucose homeostasis in diet-induced obese mice. Gut. (2014) 63:727–35. doi: 10.1136/gutjnl-2012-303839, PMID: 23804561

[33] Sokol H Pigneur B Watterlot L Lakhdari O Bermúdez-Humarán LG Gratadoux JJ . Faecalibacterium prausnitzii is an anti-inflammatory commensal bacterium identified by gut microbiota analysis of Crohn disease patients. Proc Natl Acad Sci United States America. (2008) 105:16731–6. doi: 10.1073/pnas.0804812105, PMID: 18936492 PMC2575488

[34] Sagar S Morgan ME Chen S Vos AP Garssen J van Bergenhenegouwen J . Bifidobacterium breve and Lactobacillus rhamnosus treatment is as effective as budesonide at reducing inflammation in a murine model for chronic asthma. Respir Res. (2014) 15:46. doi: 10.1186/1465-9921-15-46, PMID: 24735374 PMC4029990

[35] Gao X Lin C Feng Y You Y Jin Z Li M . Akkermansia muciniphila-derived small extracellular vesicles attenuate intestinal ischemia-reperfusion-induced postoperative cognitive dysfunction by suppressing microglia activation via the TLR2/4 signaling. Biochim Biophys Acta Mol Cell Res. (2024) 1871:119630. doi: 10.1016/j.bbamcr.2023.119630, PMID: 37967793

[36] Li Z Li M Fang X Yu D Hu X . Dietary Lactobacillus johnsonii-derived extracellular vesicles ameliorate acute colitis by regulating gut microbiota and maintaining intestinal barrier homeostasis. Food Funct. (2024) 15:11757–79. doi: 10.1039/d4fo04194a, PMID: 39545264

[37] Mandelbaum N Zhang L Carasso S Ziv T Lifshiz-Simon S Davidovich I . Extracellular vesicles of the Gram-positive gut symbiont Bifidobacterium longum induce immune-modulatory, anti-inflammatory effects. NPJ biofilms microbiomes. (2023) 9:30. doi: 10.1038/s41522-023-00400-9, PMID: 37270554 PMC10239484

[38] Kumar MA Baba SK Sadida HQ Marzooqi SA Jerobin J Altemani FH . Extracellular vesicles as tools and targets in therapy for diseases. Signal transduction targeted Ther. (2024) 9:27. doi: 10.1038/s41392-024-01735-1, PMID: 38311623 PMC10838959

[39] Padgett LE Broniowska KA Hansen PA Corbett JA Tse HM . The role of reactive oxygen species and proinflammatory cytokines in type 1 diabetes pathogenesis. Ann New York Acad Sci. (2013) 1281:16–35. doi: 10.1111/j.1749-6632.2012.06826.x, PMID: 23323860 PMC3715103

[40] Thayer TC Delano M Liu C Chen J Padgett LE Tse HM . Superoxide production by macrophages and T cells is critical for the induction of autoreactivity and type 1 diabetes. Diabetes. (2011) 60:2144–51. doi: 10.2337/db10-1222, PMID: 21715554 PMC3142064

[41] Moore F Naamane N Colli ML Bouckenooghe T Ortis F Gurzov EN . STAT1 is a master regulator of pancreatic {beta}-cell apoptosis and islet inflammation. J Biol Chem. (2011) 286:929–41. doi: 10.1074/jbc.M110.162131, PMID: 20980260 PMC3020778

[42] Roy S Pokharel P Piganelli JD . Decoding the immune dance: Unraveling the interplay between beta cells and type 1 diabetes. Mol Metab. (2024) 88:101998. doi: 10.1016/j.molmet.2024.101998, PMID: 39069156 PMC11342121

[43] Ceriello A Novials A Ortega E Canivell S La Sala L Pujadas G . Glucagon-like peptide 1 reduces endothelial dysfunction, inflammation, and oxidative stress induced by both hyperglycemia and hypoglycemia in type 1 diabetes. Diabetes Care. (2013) 36:2346–50. doi: 10.2337/dc12-2469, PMID: 23564922 PMC3714509

[44] Zamanian MY Alsaab HO Golmohammadi M Yumashev A Jabba AM Abid MK . NF-κB pathway as a molecular target for curcumin in diabetes mellitus treatment: Focusing on oxidative stress and inflammation. Cell Biochem Funct. (2024) 42:e4030. doi: 10.1002/cbf.4030, PMID: 38720663

[45] Scheithauer TPM Rampanelli E Nieuwdorp M Vallance BA Verchere CB van Raalte DH . Gut Microbiota as a Trigger for Metabolic Inflammation in Obesity and Type 2 Diabetes. Front Immunol. (2020) 11:571731. doi: 10.3389/fimmu.2020.571731, PMID: 33178196 PMC7596417

[46] Rosendo-Silva D Viana S Carvalho E Reis F Matafome P . Are gut dysbiosis, barrier disruption, and endotoxemia related to adipose tissue dysfunction in metabolic disorders? Overview of the mechanisms involved. Intern Emerg Med. (2023) 18:1287–302. doi: 10.1007/s11739-023-03262-3, PMID: 37014495 PMC10412677

[47] Acosta-Montaño P Rodríguez-Velázquez E Ibarra-López E Frayde-Gómez H Mas-Oliva J Delgado-Coello B . Fatty Acid and Lipopolysaccharide Effect on Beta Cells Proteostasis and its Impact on Insulin Secretion. Cells. (2019) 8(8):884. doi: 10.3390/cells8080884, PMID: 31412623 PMC6721695

[48] Lu J Zhang C Li L Xue W Zhang C Zhang X . Unique Features of Pancreatic-Resident Regulatory T Cells in Autoimmune Type 1 Diabetes. Front Immunol. (2017) 8:1235. doi: 10.3389/fimmu.2017.01235, PMID: 29033948 PMC5626883

[49] Bettini M Bettini ML . Function, Failure, and the Future Potential of Tregs in Type 1 Diabetes. Diabetes. (2021) 70:1211–9. doi: 10.2337/dbi18-0058, PMID: 34016597 PMC8275894

[50] Shimojima Y Kishida D Ichikawa T Takamatsu R Nomura S Sekijima Y . Oxidative Stress Promotes Instability of Regulatory T Cells in Antineutrophil Cytoplasmic Antibody-Associated Vasculitis. Front Immunol. (2021) 12:789740. doi: 10.3389/fimmu.2021.789740, PMID: 34950150 PMC8691772

[51] Round JL Lee SM Li J Tran G Jabri B Chatila TA . The Toll-like receptor 2 pathway establishes colonization by a commensal of the human microbiota. Sci (New York NY). (2011) 332:974–7. doi: 10.1126/science.1206095, PMID: 21512004 PMC3164325

[52] Wang L Liang Y Zhao C Ma P Zeng S Ju D . Regulatory T cells in homeostasis and disease: molecular mechanisms and therapeutic potential. Signal transduction targeted Ther. (2025) 10:345. doi: 10.1038/s41392-025-02326-4, PMID: 41087343 PMC12521743

[53] Burrack AL Martinov T Fife BT . T Cell-Mediated Beta Cell Destruction: Autoimmunity and Alloimmunity in the Context of Type 1 Diabetes. Front Endocrinol (Lausanne). (2017) 8:343. doi: 10.3389/fendo.2017.00343, PMID: 29259578 PMC5723426

[54] Onishi Y Fehervari Z Yamaguchi T Sakaguchi S . Foxp3+ natural regulatory T cells preferentially form aggregates on dendritic cells *in vitro* and actively inhibit their maturation. Proc Natl Acad Sci United States America. (2008) 105:10113–8. doi: 10.1073/pnas.0711106105, PMID: 18635688 PMC2481354

