# Supplementary figures and images for: Extracellular vesicles from Akkermansia muciniphila protect streptozotocin-induced diabetic mice by regulating glucose homeostasis, oxidative stress, and immune tolerance
Source: Front Immunol. 2026 Apr 7;17:1739048. doi: 10.3389/fimmu.2026.1739048 (PMC13095594; doi:10.3389/fimmu.2026.1739048)

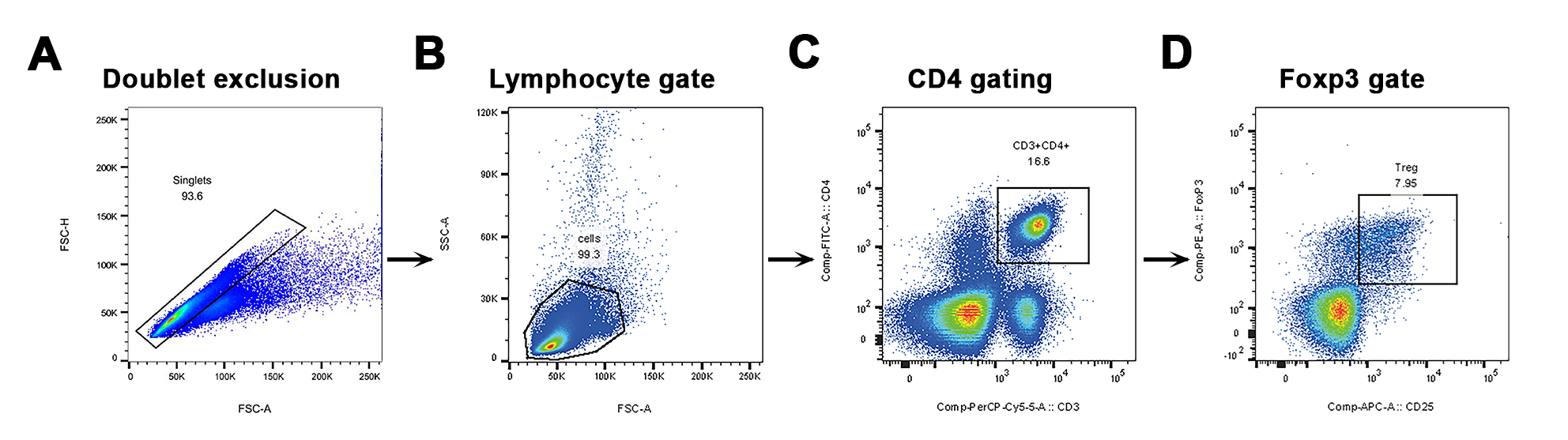

Supplement: Supplementary file 1 [file Image1.tif]

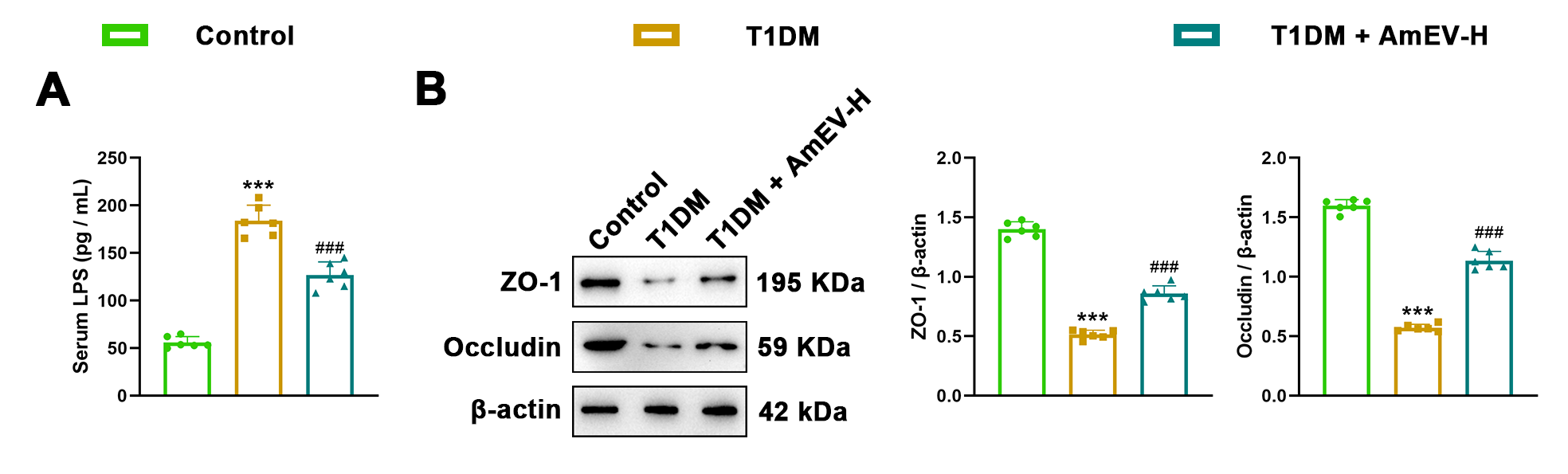

Supplement: Supplementary file 2 [file Image2.tif]
